# Supplementary material for: Poly(carbazole-co-1,4-dimethoxybenzene): Synthesis, Electrochemiluminescence Performance, and Application in Detection of Fe3+
Source: Polymers (Basel). 2022 Jul 27;14(15):3045. doi: 10.3390/polym14153045 (PMC9370792; doi:10.3390/polym14153045)
Supplement: Supplementary file 1 [file polymers-14-03045-s001.zip › polymers-1777962-supplementary.pdf]

Supplementary Materials: Poly(carbazole-co-1,4-dimethoxybenzene): Synthesis, Electrochemiluminescence Performance, and Application in Detection of Fe^{3+}

Pengchong Hou, Xian Zhang, Qian Lu, Shunwei Chen, Qiang Liu, Congde Qiao and Hui Zhao

1. Synthetic materials and methods

1.1. Synthesis of 9-alkylcarbazole

A mixture of KOH powder (13 g, 0.23 mol) and carbazole (6.6 g, 0.04 mol) in DMF (65 mL) was stirred in a three-necked bottle at room temperature. Then, bromoethane (6.54 g, 0.06 mol) was dissolved in DMF (30 mL), and dripped slowly for about 30 minutes. Then, the mixture was stirred for 12 hours and added to water. After being filtered and dried, a pale yellow solid was obtained. Finally, the resulting product was purified by recrystallization twice to obtain 7.15 g of white rod-shaped crystals (yield: 91.6%). The ^{1}H NMR (400 MHz, $CDCl_3$) with δ (ppm): 8.12(d, *J* =7.1 Hz, 2H)，7.39(m, 2H), 7.37(d, *J* =6.2 Hz, 2H), 7.20(m, 2H), 4.29(q, 2H), 1.39(t, *J* =7.1 Hz, 3H).

A similar synthesis process was used to obtain 9-butylcarbazole, 9-octylcarbazole, and 9-hexadecylcarbazole. The ^{1}H NMR (400 MHz, $CDCl_3$) of 9-butylcarbazole with δ (ppm): 8.14 (d, *J* =7.6 Hz, 2H), 7.68 (d, *J* =8.5 Hz, 2H), 7.41 (m, 2H), 7.21 (m, 2H), 4.36 (t, *J* =6.9 Hz, 2H), 1.72 (m ,2H), 1.43 (m, 2H), 0.86 (t, *J* =7.2 Hz, 3H). The ^{1}H NMR (400 MHz, DMSO) of 9-octylcarbazole with δ (ppm): 8.09 (d, *J* = 7.2 Hz, 2H), 7.56 (m, 2H), 7.49 (m, 2H), 7.15 (d, *J* = 7.6 Hz, 2H), 4.31 (t, *J* =12.2 Hz, 2H), 1.69 (m, 2H), 1.17-1.10 (m, 10H), 0.79 (t, *J* =6.4 Hz, 3H). The ^{1}H NMR (400 MHz, $CDCl_3$) of 9-hexadecylcarbazole with δ (ppm): 11.18 (s, 2H), 7.70-7.59 (m, 8H), 4.27 (m, 2H), 1.69 (m, 2H), 1.17-1.10 (m, 28H), 0.79 (t, *J* =6.4 Hz, 3H).

1.2. Synthesis of 3,6-Diformyl-N-alkylcarbazole

DMF (30 mL), 1,2-dichloroethane (25 mL), and $POCl_3$ (17 mL) were added to a 250 mL three-necked flask and stirred below 0 °C until the solution solidified, then the mixture was shifted from the cold-water bath to an oil bath and dissolved at room temperature. Afterward, *N*-ethylcarbazole (4.3 g, 22.02 mmol) was added to the mixed solution, then the temperature was raised to 100 °C and reacted for 60 hours. The obtained dark brown viscous substance was added to ice water and hydrolyzed to stir for 1 hour. At the same time, the pH of the mixture was adjusted to neutral with NaOH (2 mol/L), and the red-brown precipitate was found. The filtered residue was dissolved in dichloromethane and washed with water, then magnesium sulfate was added to remove H_2O, and the desired product was obtained by rotary evaporation. The product was extracted by silica gel column chromatography using petroleum ether: dichloromethane=1:2 as elution. Finally, yellow-green solids were obtained with a yield of 83% (4.62 g). Mp of M1 is 113 ℃. The ^{1}H NMR (400 MHz, $CDCl_3$) is shown in Figure S1 with δ (ppm): 10.13 (t, *J* =6.7 Hz, 2H), 8.61 (m, 2H), 8.09 (m, 2H), 7.58 (m, 2H), 4.47 (q, *J* =8.4 Hz, 2H), 1.25 (t, *J* =7.2 Hz, 3H).

A similar synthesis process was used to obtain M2, M3, M4. The Mp values of M2-M4 are 104 ℃, 94 ℃, and 91 ℃. The ^{1}H NMR (400 MHz, DMSO) of M2 is shown in Figure S2 with δ (ppm): 10.05 (m, 2H), 8.77 (s, 2H), 8.06 (d, *J* =9.6 Hz, 2H), 7.89 (d, *J* =8.4 Hz, 2H), 4.52 (t, *J* =7.8 Hz, 2H), 1.80 (m, 2H), 1.38 – 1.19 (m, 2H), 0.87 (t, *J* = 7.3 Hz, 3H). The ^{1}H NMR (400 MHz, DMSO) of M3 is shown in Figure S3 with δ (ppm): 10.12 (s, 2H), 8.91 (s, 2H), 8.07 (d, *J* =8.5 Hz, 2H), 7.82 (t, *J* =7.8 Hz, 2H), 4.49 (t, *J* =8.5 Hz, 2H), 1.78 (m, 2H), 1.25-1.16 (m, 10H), 0.79 (t, *J* =6.4 Hz, 3H). The ^{1}H NMR (400 MHz, DMSO) of M4 is shown in Figure S4 with δ (ppm): 9.88 (s, 2H), 8.72 (s, 2H), 8.24 (d, *J* = 7.3 Hz, 2H), 7.78 (d, *J* = 8.4 Hz, 2H), 4.45 (t, *J* = 6.8 Hz, 2H), 1.78 (m, 2H), 1.25-1.16 (m, 26 H), 0.79 (t, *J* =6.4 Hz, 3H).

1.3. Synthesis of M5

1,4-dioxane (50 mL), 1,4-dimethoxybenzene (10 g, 72.38 mmol), and concentrated hydrochloric acid (10 mL) were added to a three-necked flask of 250 mL, and HCl gas was continuously introduced into it. At the same time, the temperature was slowly raised to 60 °C. Next, 10 mL of the formaldehyde solution divided equally into three was added to the mixture solution. The reaction was carried out for 3 hours under the magnetic stirring, and the excess HCl gas was treated by NaOH (0.1 mol/L). Then, concentrated hydrochloric acid (10 mL) and HCHO (10 mL) were added to the reaction vessel, and the reaction was continued for 1 hour. Then, the white reaction intermediate was obtained by recrystallization with dimethyl ketone. The ^{1}H NMR (400 MHz, DMSO) with δ (ppm): 7.16 (s, 2H), 4.60 (t, *J*=9.6 Hz, 4H), 3.81 (s, 6H).

The reaction intermediate (3.5 g, 14.89 mmol) and triethyl phosphite (18 mL) were added to a 100 mL volumetric flask. The temperature was gradually raised to 90 °C under a nitrogen atmosphere, and the solvent was cooled and refluxed under a nitrogen atmosphere for 24 h. The pure white precipitate was lyophilized and further filtered to obtain the crude product. The product was extracted with chloroform and then dried with anhydrous $MgSO_4$. The impurities were filtered out by vacuum, and the solvent was evaporated and washed with n-hexane to obtain the pure white expected substance (Mp=115 ℃) with a yield of 75%. The ^{1}H NMR (400 MHz, DMSO) is shown in Figure S5 with δ (ppm): 6.88 (s, 2H), 3.92 (q, 8H), 3.73 (s, 6H), 1.16 (t, *J*=7.0 Hz, 12H).

1.4. Synthesis of PCMB-D

M5 (0.80 g, 1.83 mmol) was added to a three-necked flask of 50 mL in a nitrogen atmosphere below 0 °C. THF (15 mL) containing potassium tert-butoxide (t-BuOK) (1.1 g, 11.45 mmol) was slowly added, and it is normal that the color changes quickly. After stirring for 20 minutes, the reaction device was moved to an oil bath. M1 (0.56 g, 2.23 mmol) dissolved in 10 mL of THF solution (10 mL) was injected into the mixed solution and reacted for 48 h at room temperature. Then, the reaction product was poured into methanol (300 mL) with constant stirring, and the precipitate was filtered. The filter residue was centrifuged multiple times with CH_3OH, and then the solvent was removed and dried to obtain a yellow solid substance. The ^{1}H NMR (400 MHz, DMSO) of P-1 is shown in Figure S6 with δ (ppm): 8.57 – 8.04 (m, 2H), 7.79 – 7.26 (m, 8H), 7.13 – 6.50 (m, 2H), 4.36 (d, *J* = 16.2 Hz, 2H), 3.96 (s, 6H), 1.60 – 0.65 (t, *J* =6.3 Hz, 3H). The ^{13}C NMR (101 MHz, DMSO) of P-1 is shown in Figure S7 with δ (ppm): 151.36, 140.20, 129.53, 123.30, 56.60, 14.33. IR (KBr) ν/cm^{-1} of P-1: 3044 cm^{-1} ($C\text{-}H_{ol}$); 2933 cm^{-1} ($C\text{-}H_{al}$)； 1593 cm^{-1} ($C{=}C_{ar}$)， 1479 cm^{-1} ($C\text{-}H_{ar}$)； 1207 cm^{-1} (C-O)； 1037 cm^{-1} (C-N)； 792 cm^{-1}($C{=}C_{ol}$).

The same synthetic process was carried out to obtain P-2, P-3, and P-4. The ^{1}H NMR (400 MHz, $CDCl_3$) of P-2 is shown in Figure S8 with δ (ppm): 8.73 – 7.89 (m, 2H), 7.84 – 7.33 (m, 10H), 6.71 (d, *J* = 8.3 Hz, 1H), 5.06 (s, 2H), 4.01 (dd, *J* = 16.5, 5.8 Hz, 2H), 3.96 (s, 6H), 3.26 (s, 2H), 2.24 – 1.56 (m, 2H), 1.49 – 0.97 (t, *J* = 8.2 Hz, 3H). The ^{13}C NMR (101 MHz, $CDCl_3$) of P-2 is shown in Figure S9 with δ (ppm): 77.36, 77.04, 76.72, 31.78, 29.17, 29.03, 27.26, 22.59, 14.04. IR (KBr) ν/cm^{-1} of P-2: 3042 cm^{-1} ($C\text{-}H_{ol}$); 2925 cm^{-1} ($C\text{-}H_{al}$)； 1591 cm^{-1} ($C{=}C_{ar}$)， 1498 cm^{-1} ($C\text{-}H_{ar}$)； 1212 cm^{-1} (C-O)； 1046 cm^{-1} (C-N)； 796 cm^{-1}($C{=}C_{ol}$). The ^{1}H NMR (400 MHz, $CDCl_3$) of P-3 was shown in Figure S10 with δ (ppm): 8.32 (s, 2H), 7.72 (s, 2H), 7.60 (dd, *J* = 8.6, 7.8 Hz, 2H), 7.36 (s, 4H), 6.72 (d, *J* = 5.7 Hz, 2H), 4.50 – 4.15 (m, 2H), 3.94 (s, 6H), 2.13 – 1.65 (m, 2H), 1.27 (m, 10H), 0.88 (t, *J* = 9.2 Hz, 3H). The ^{13}C NMR (101 MHz, $CDCl_3$) of P-3 is shown in Figure S11 with δ (ppm): 77.35, 77.04, 76.72, 56.40, 31.79, 29.37, 29.19, 29.03, 27.27, 22.60, 14.07. IR (KBr) ν/cm^{-1} of P-3: 3046 cm^{-1} ($C\text{-}H_{ol}$); 2927 cm^{-1} ($C\text{-}H_{al}$)； 1600 cm^{-1} ($C{=}C_{ar}$)， 1485 cm^{-1} ($C\text{-}H_{ar}$)； 1208 cm^{-1} (C-O)； 1044 cm^{-1} (C-N)； 798 cm^{-1}($C{=}C_{ol}$). The ^{1}H NMR (400 MHz, $CDCl_3$) of P-4 is shown in Figure S12 with δ (ppm): 8.42 – 7.83 (m, 2H), 7.67 (s, 2H), 7.57 (d, *J* = 9.1 Hz, 2H), 7.35 (s, 2H), 6.71 (d, *J* = 8.1 Hz, 1H), 4.43 – 3.63 (m, 5H), 4.33 (d, *J* = 8.8 Hz, 2H), 4.10 – 3.57 (m, 3H), 2.95 – 2.32 (m, 2H), 2.24 – 0.95 (m, 12H), 0.87 (s, 3H). The ^{13}C NMR (101 MHz, $CDCl_3$) of P-4 is shown in Figure S13 with δ (ppm): 77.35, 77.32, 77.03, 77.00, 76.71, 76.68, 31.75, 30.94, 30.85, 29.33, 29.00, 25.59, 22.53, 14.00. IR (KBr) ν/cm^{-1} of P-4: 3048 cm^{-1} ($C\text{-}H_{ol}$); 2933 cm^{-1} ($C\text{-}H_{al}$)； 1593 cm^{-1} ($C{=}C_{ar}$)， 1482 cm^{-1} ($C\text{-}H_{ar}$)； 1210 cm^{-1} (C-O)； 1037 cm^{-1} (C-N)； 798 cm^{-1}($C{=}C_{ol}$).

2. NMR spectra of materials

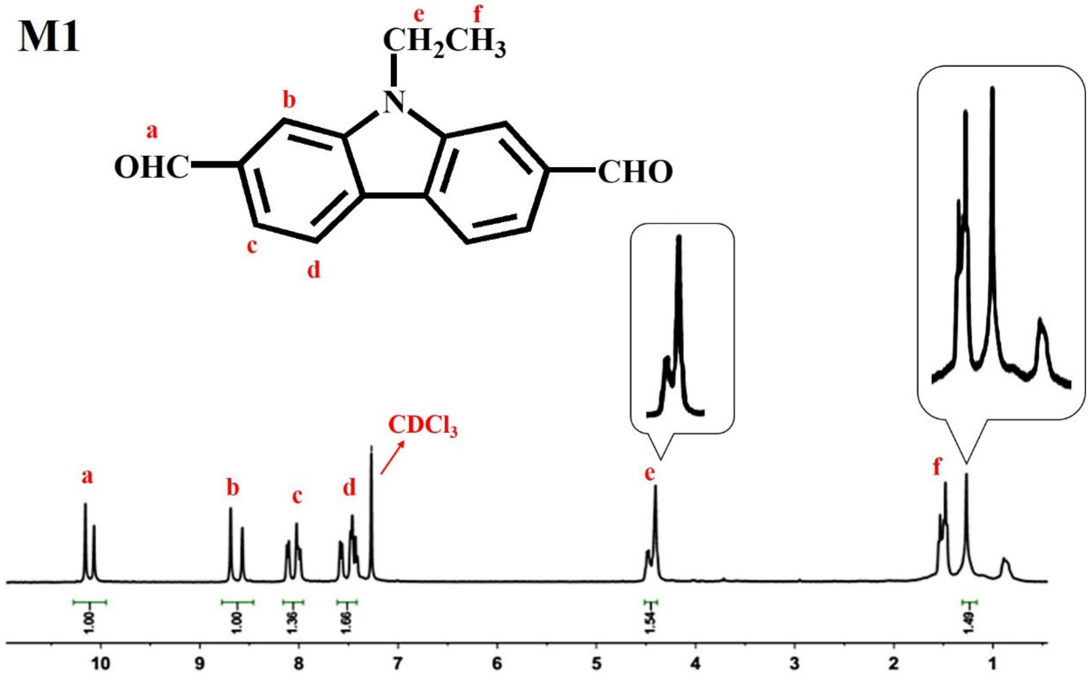

Figure S1. ^{1}H NMR spectrum of M1 in $CDCl_3$ at 298 K.

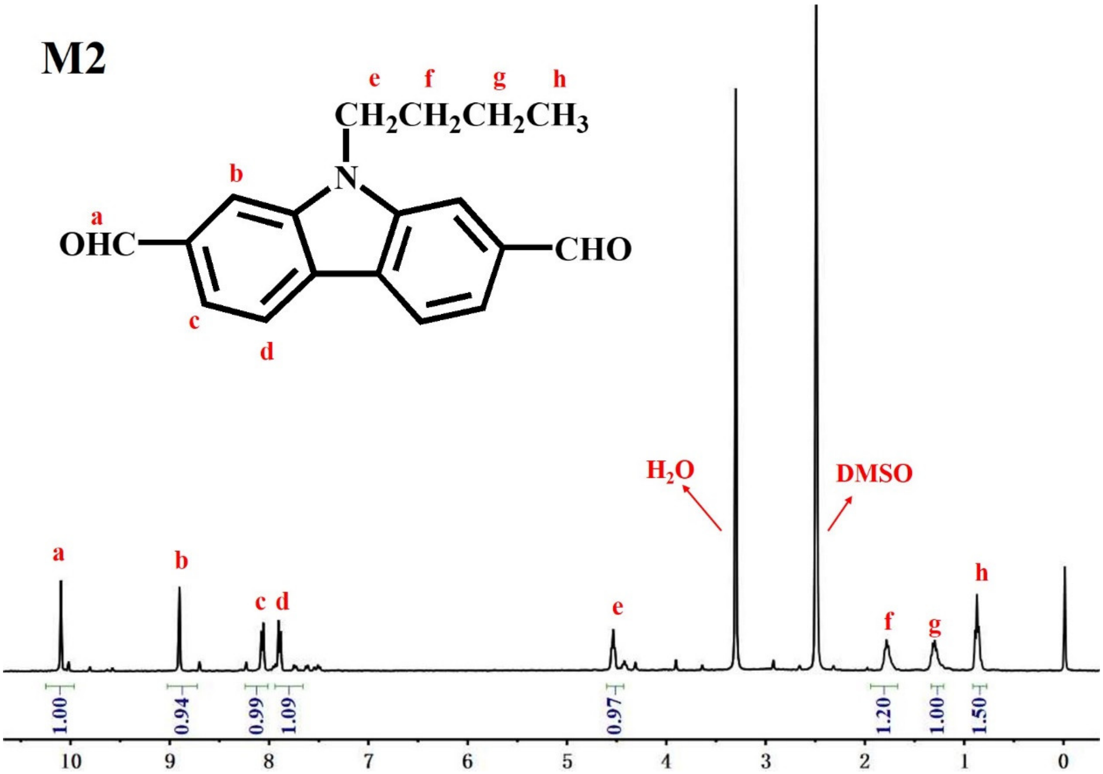

Figure S2. ^{1}H NMR spectrum of M2 in DMSO at 298 K.

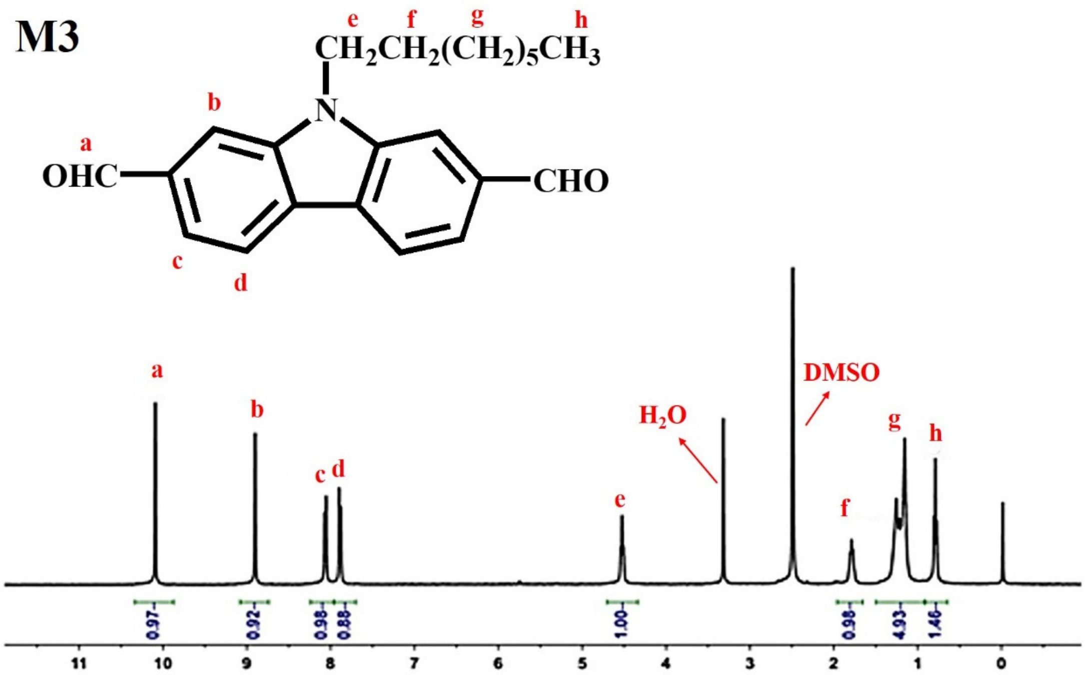

Figure S3. ^{1}H NMR spectrum of M3 in DMSO at 298 K.

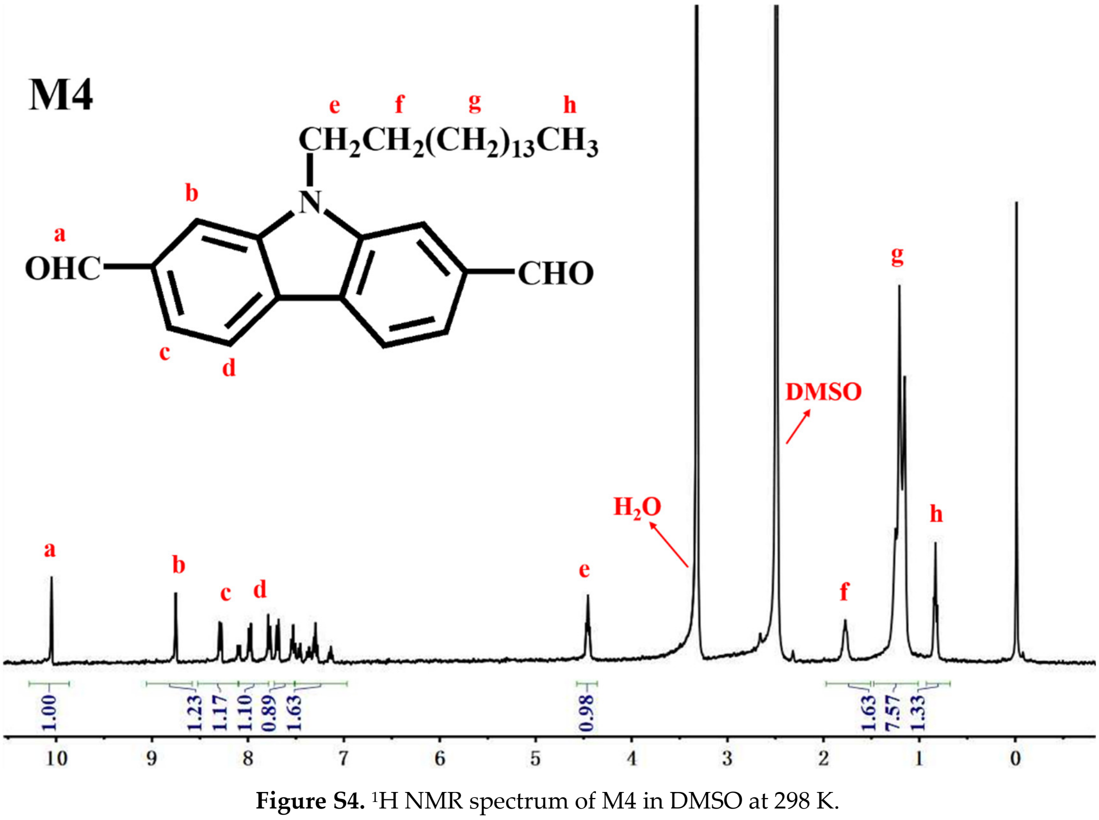

Figure S4. ^{1}H NMR spectrum of M4 in DMSO at 298 K.

M5

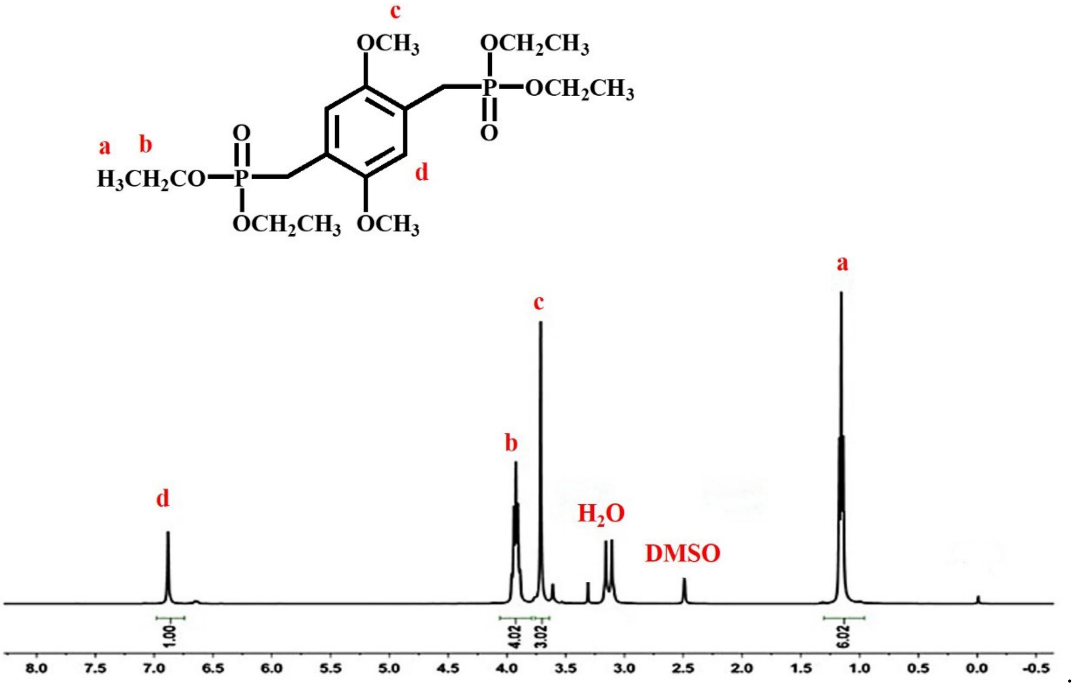

Figure S5. ^{1}H NMR spectrum of M5 in DMSO at 298 K.

P-1

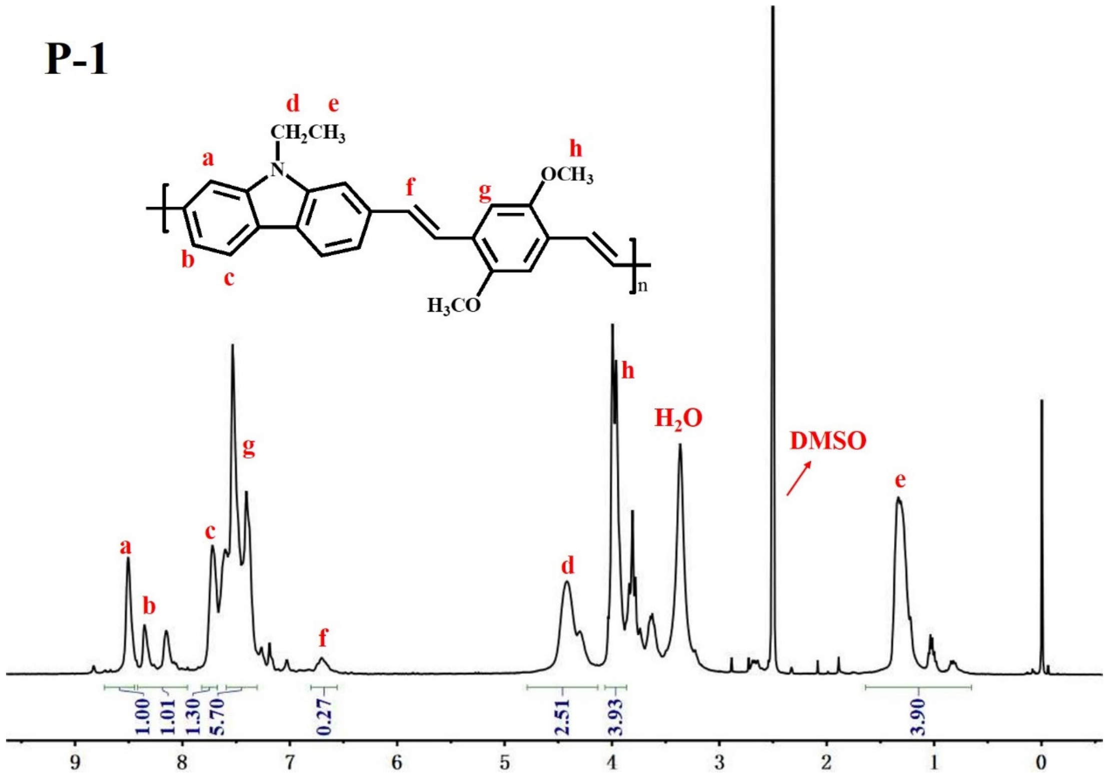

Figure S6. ^{1}H NMR spectrum of P-1 in DMSO at 298 K.

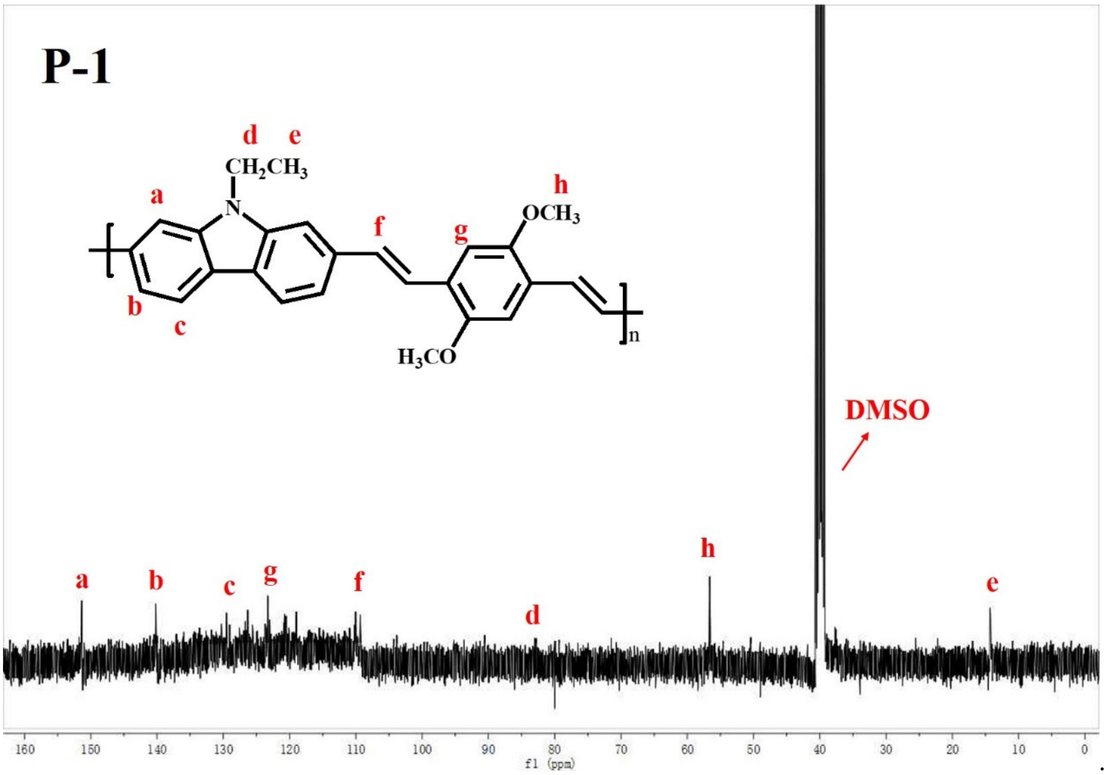

Figure S7. ^{13}C NMR spectrum of P-1 in DMSO at 298 K.

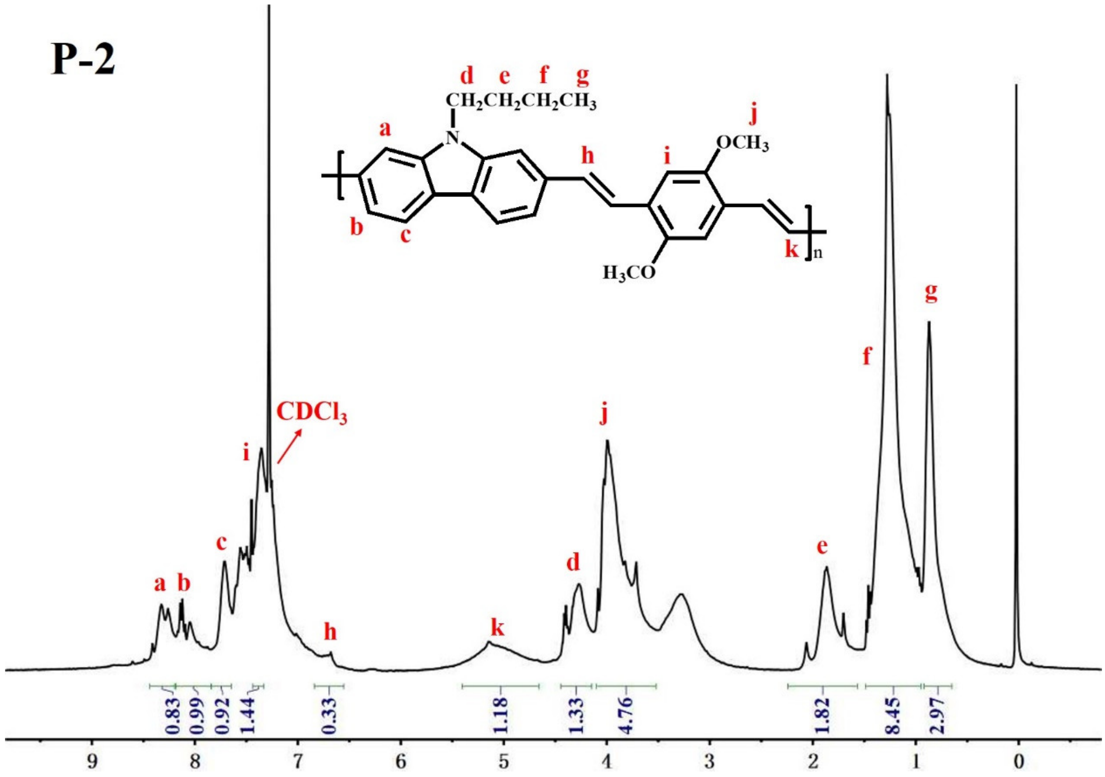

Figure S8. ^{1}H NMR spectrum of P-2 in $CDCl_3$ at 298 K.

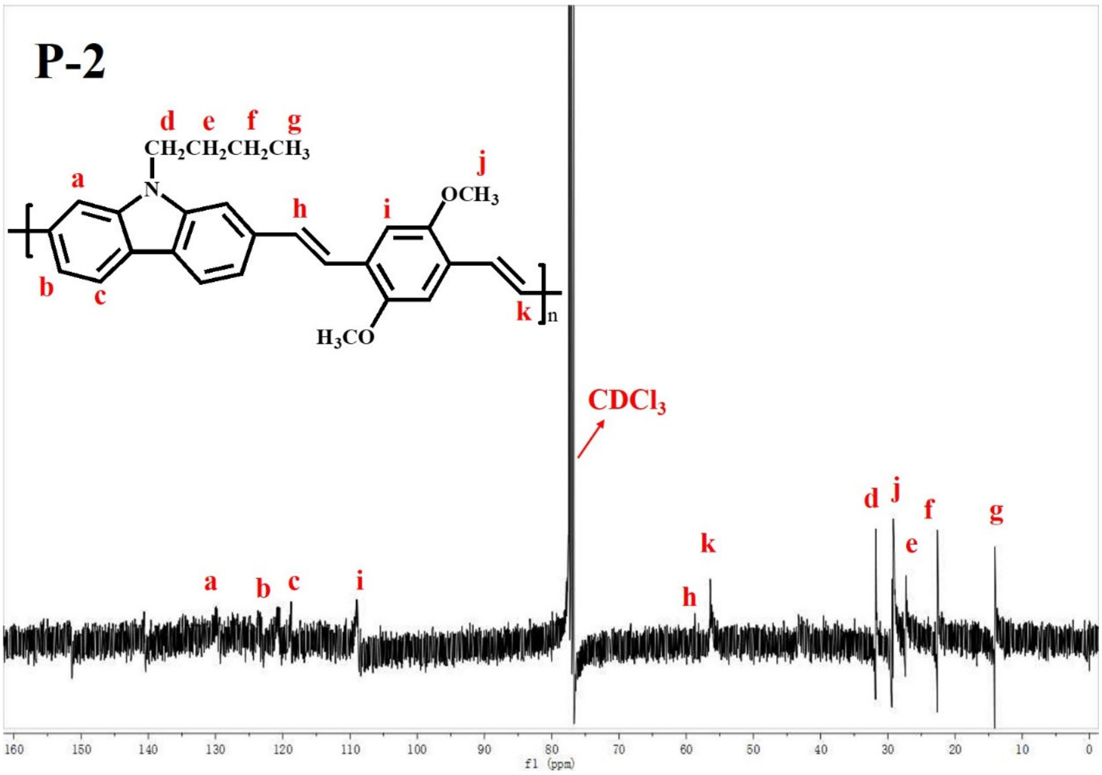

Figure S9. ^{13}C NMR spectrum of P-2 in $CDCl_3$ at 298 K.

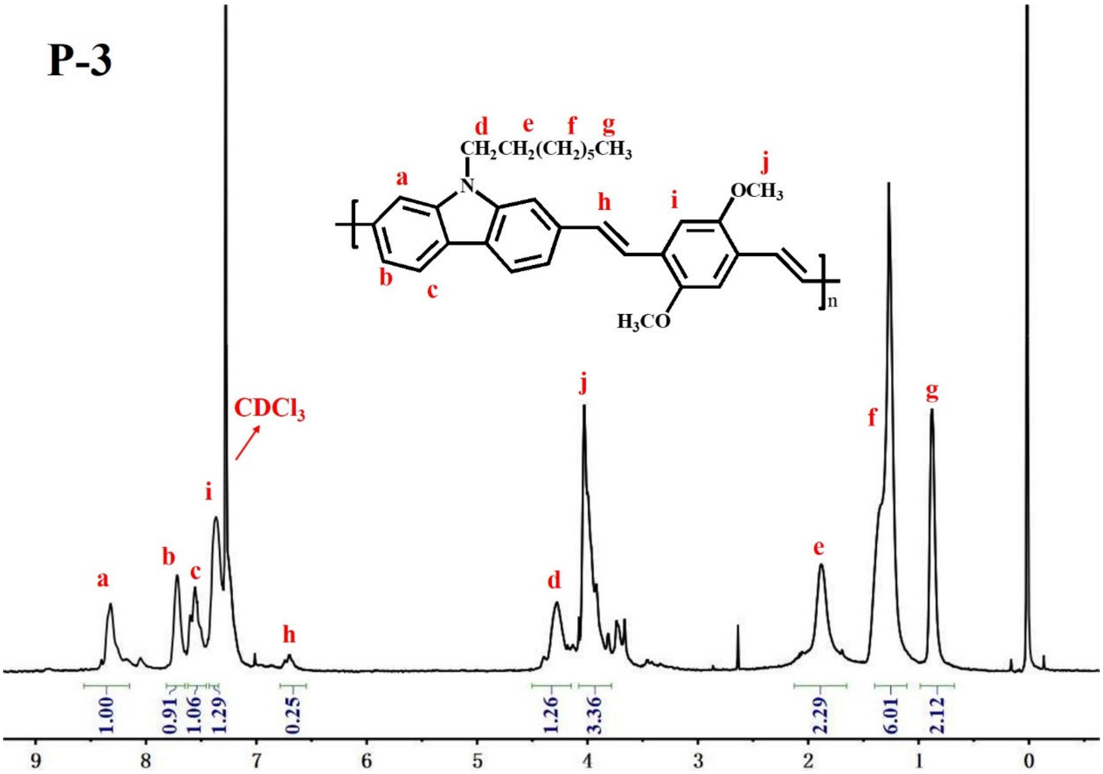

Figure S10. ^{1}H NMR spectrum of P-3 in $CDCl_3$ at 298 K.

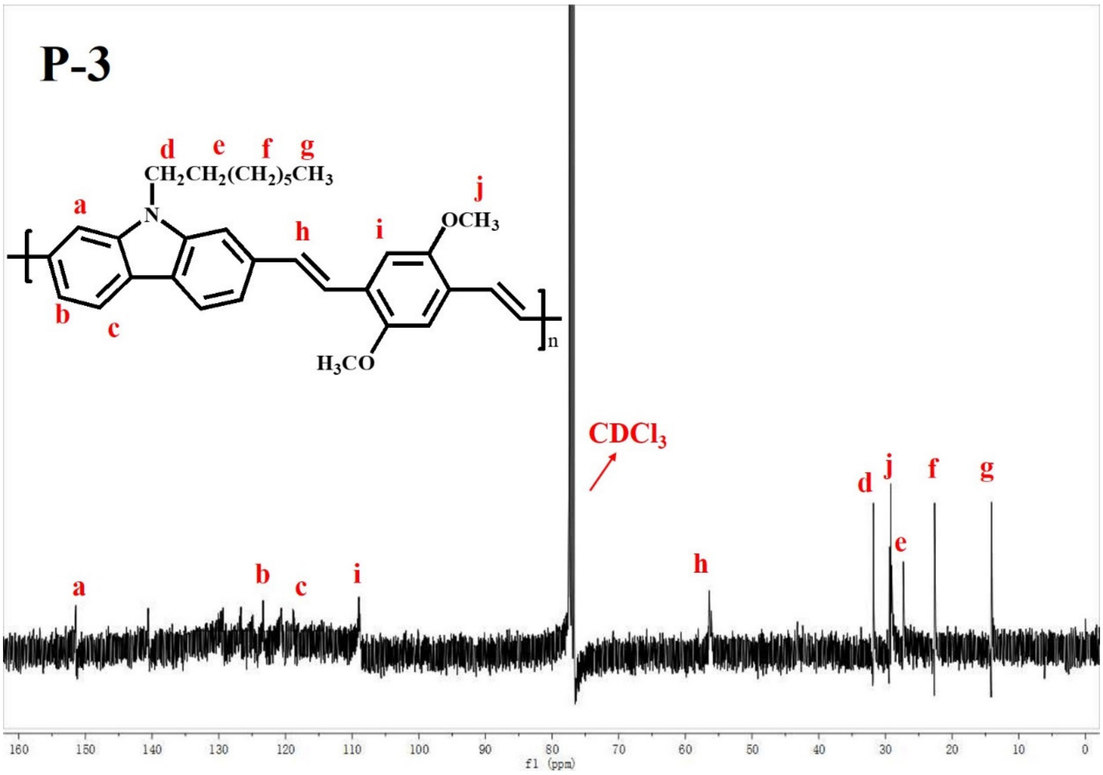

Figure S11. ^{13}C NMR spectrum of P-3 in $CDCl_3$ at 298 K.

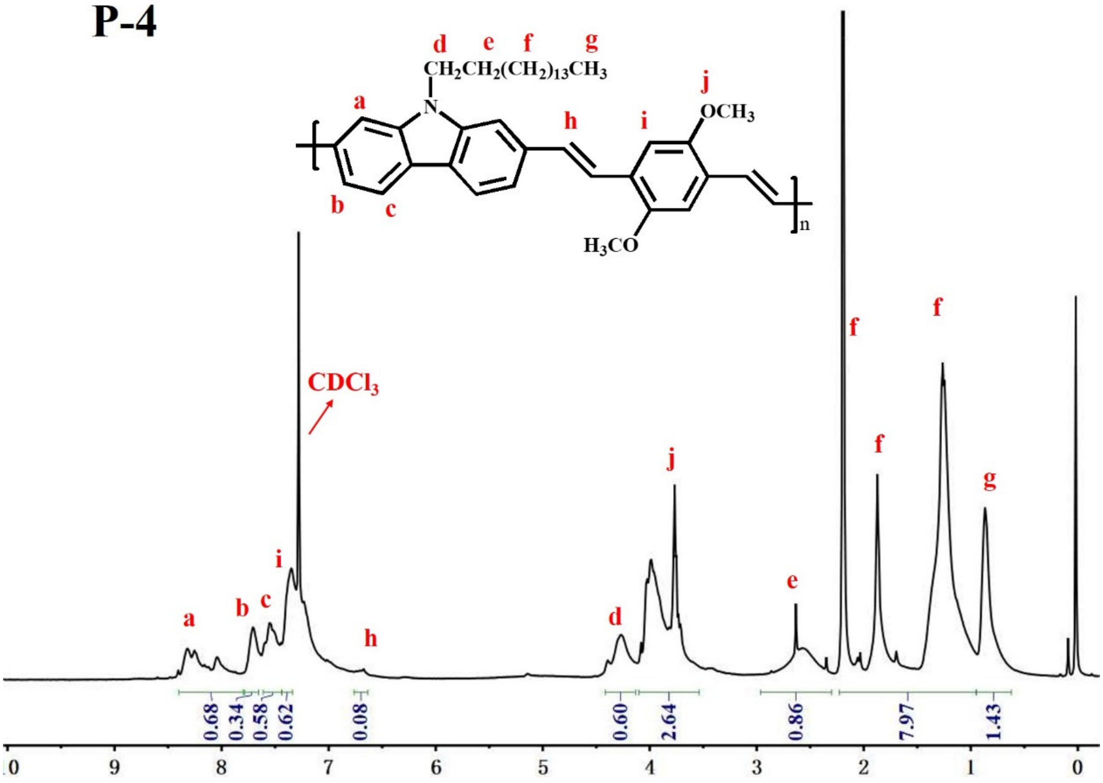

Figure S12. ^{1}H NMR spectrum of P-4 in $CDCl_3$ at 298 K.

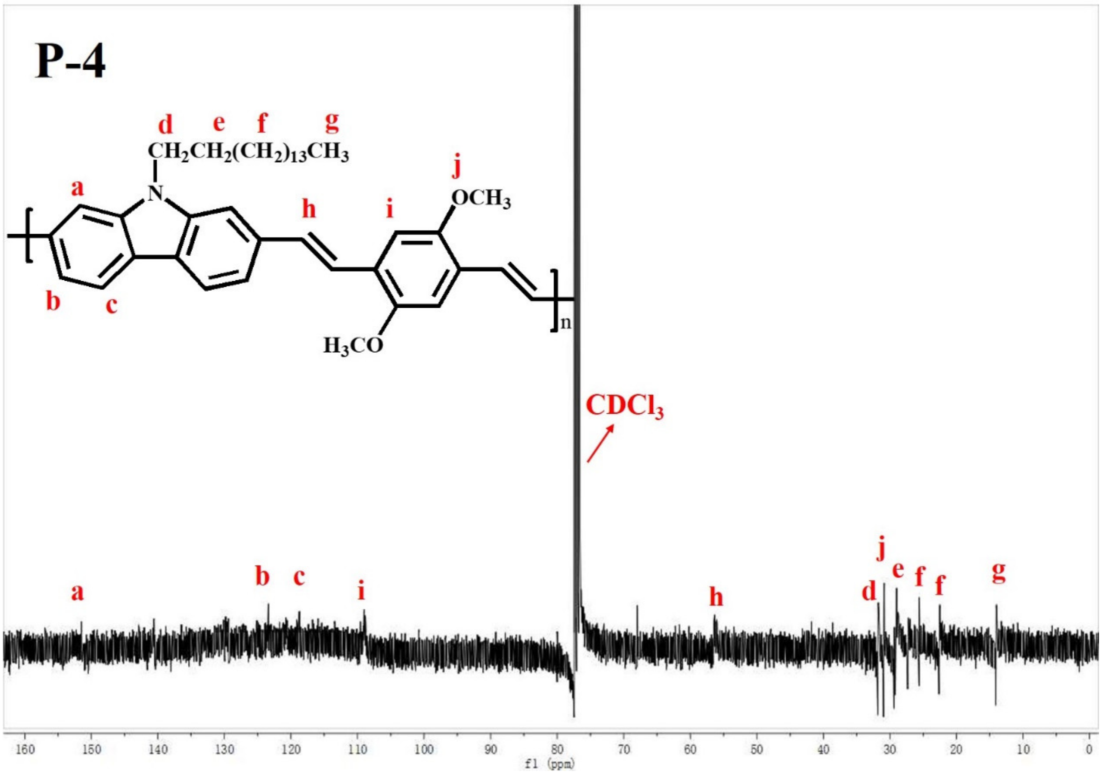

Figure S13. ^{13}C NMR spectrum of P-4 in $CDCl_3$ at 298 K.

3. FTIR spectra of PCMB-D

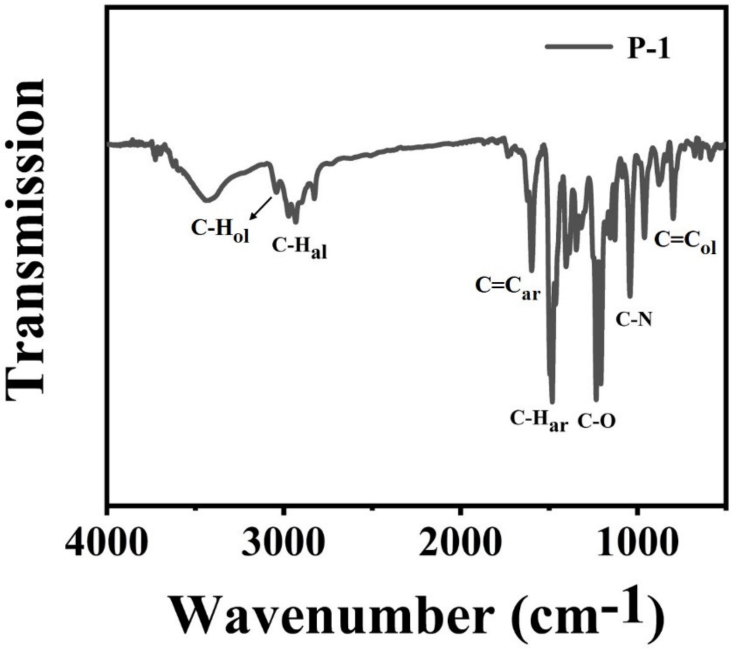

Figure S14. FTIR spectrum of P-1 (al, ar, and ol are the abbreviations of alkyl, aromatic ring, and olefin, respectively).

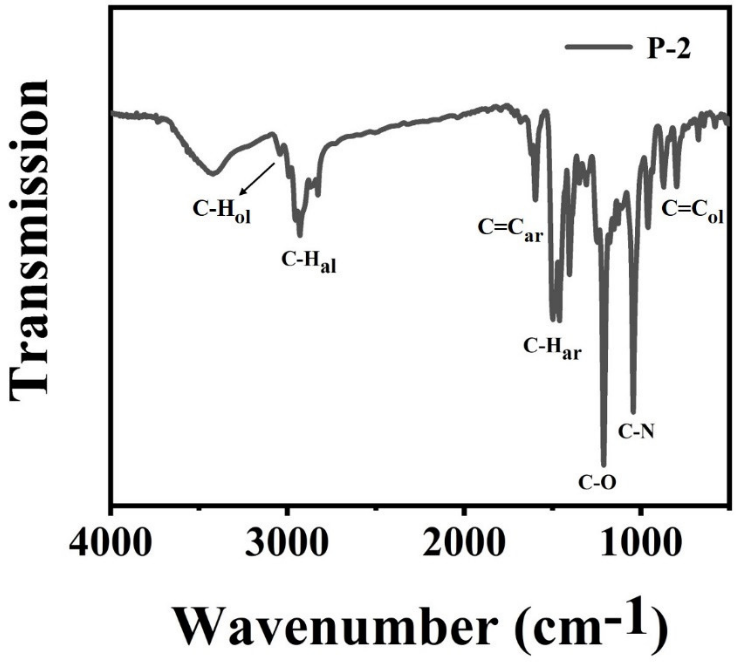

Figure S15. FTIR spectrum of P-2.

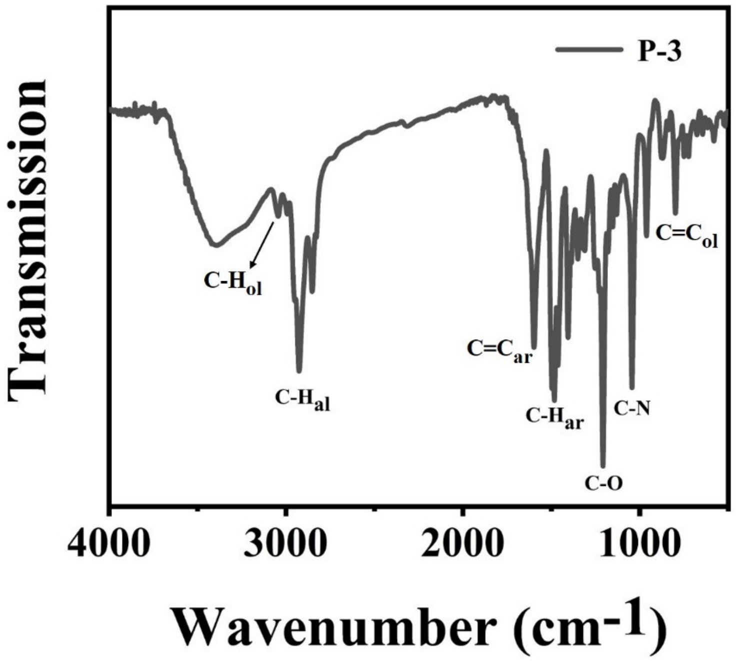

Figure S16. FTIR spectrum of P-3.

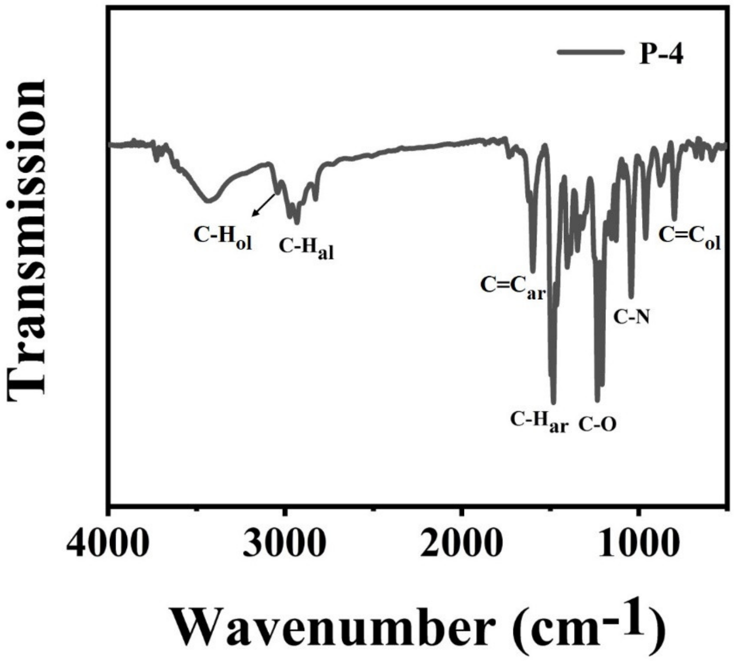

Figure S17. FTIR spectrum of P-4.

4. Solvation effect of PCMB-D

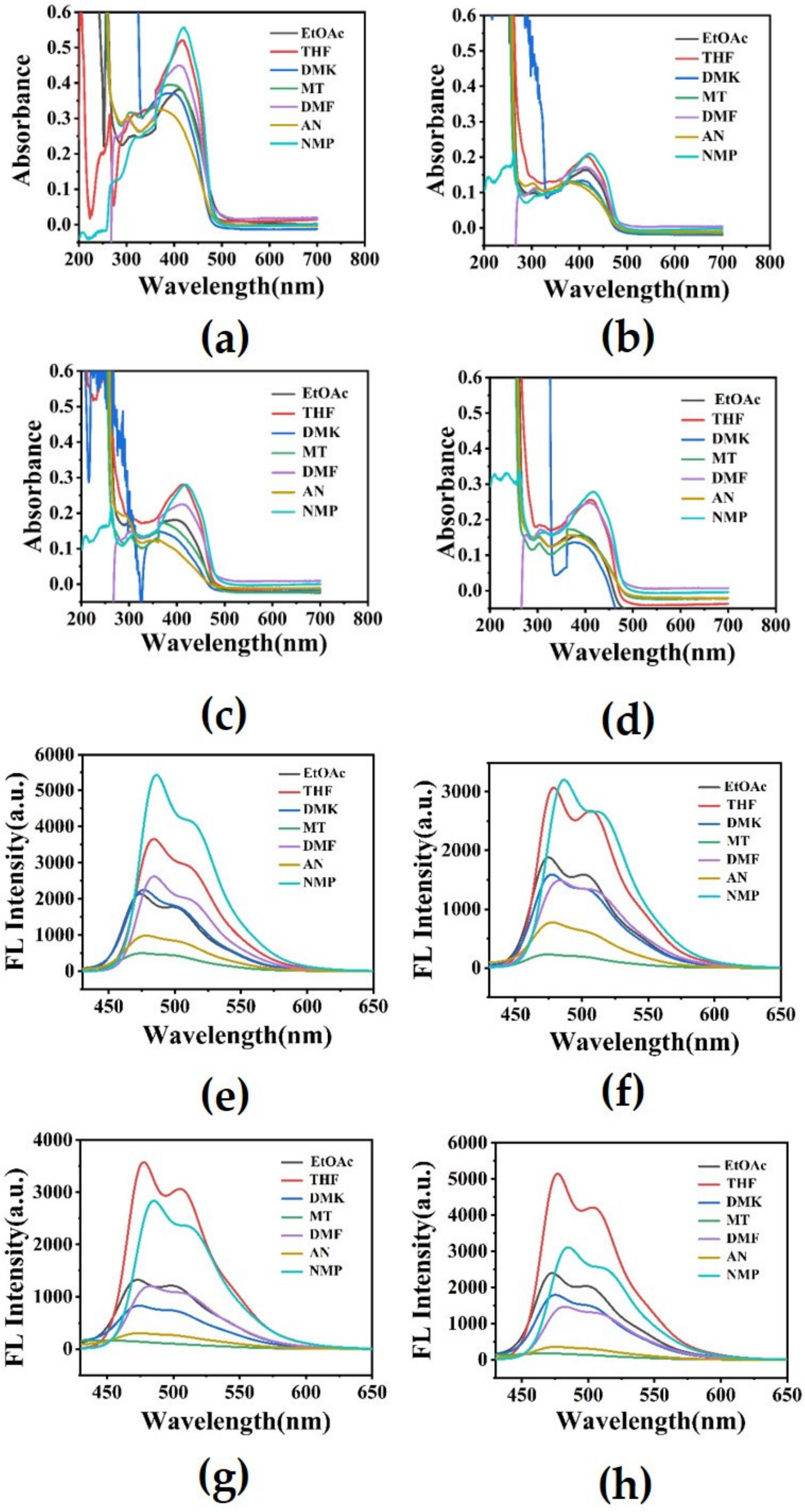

Figure S18. (**a**) Absorption spectra of P-1; (**b**) absorption spectra of P-2; (**c**) absorption spectra of P-3; (**d**) absorption spectra of P-4; (**e**) emission spectra of P-1; (**f**) emission spectra of P-2; (**g**) emission spectra of P-3; (**h**) emission spectra of P-4 in different polarity solvents.

5. P-3 in NMP under sunlight and UV light

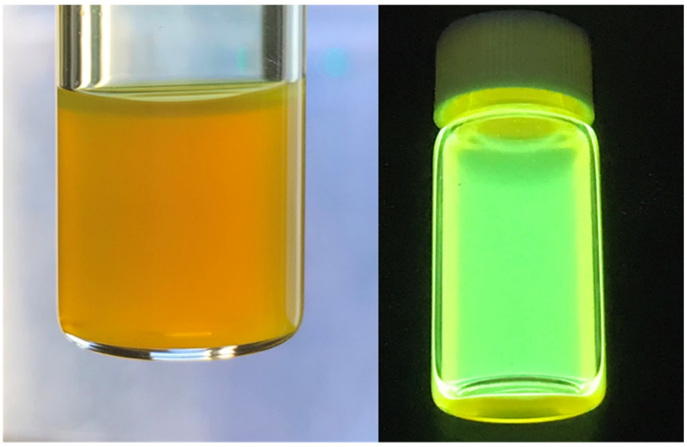

(a) **(b)**

Figure S19. (**a**) Photograph of P-3 in NMP under sunlight; (**b**) photograph of P-3 in NMP under UV light (365nm).

6. Aggregation effect of P-3

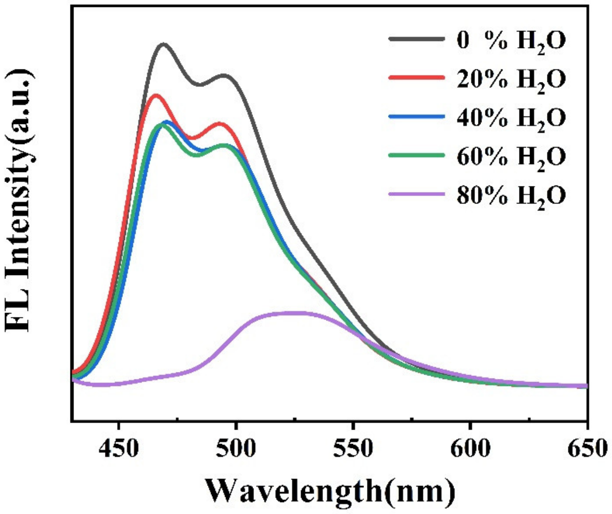

Figure S20. Fluorescence intensity of PCMB-D in mixed solution of water and NMP, and the proportion of water in the mixed solution is from 0% to 80%.

7. TGA curves of PCMB-D

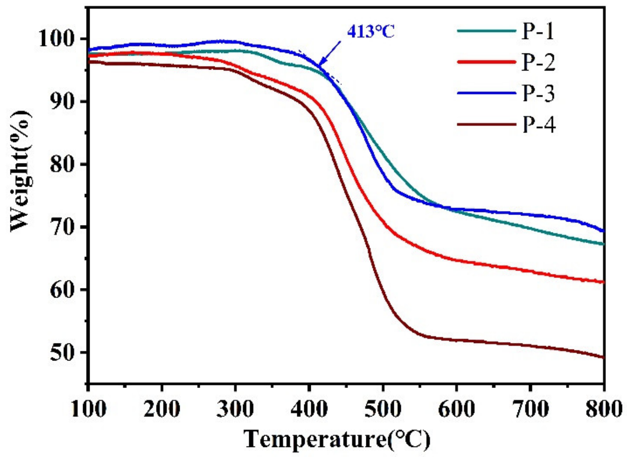

Figure S21. TGA curves of P-1, P-2, P-3, and P-4 in the presence of nitrogen.

8. TGA curves of PCMB-D

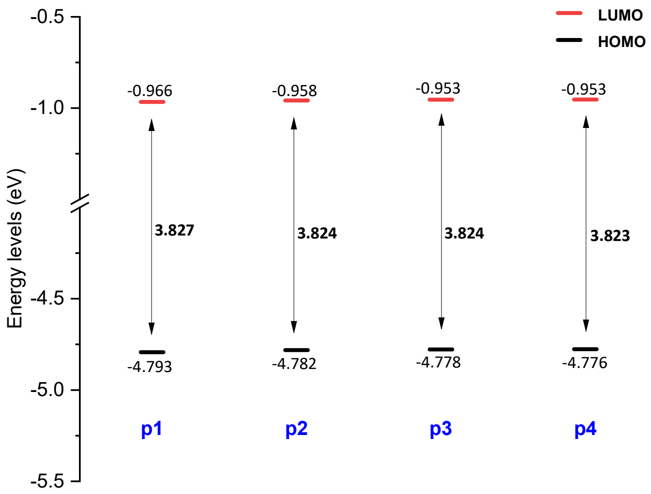

Figure S22. Calculated HOMO–LUMO enegy levels for structural models of P-1, P-2, P-3, and P-4.
